# Patient‐present teaching in the clinic: Effect on agency and professional behaviour

**DOI:** 10.1111/medu.14623

**Published:** 2021-09-06

**Authors:** Bavenjit Cheema, Meredith Li, Daniel Ho, Erica Amari, Heather Buckley, Carolyn Canfield, Cary Cuncic, Laura Nimmon, Anneke Van Enk, Kiran Veerapen, Katherine M. Wisener, Cheryl Lynn Holmes

**Affiliations:** ^1^ Faculty of Medicine University of British Columbia Vancouver British Columbia Canada; ^2^ Office of Faculty Development, Faculty of Medicine University of British Columbia Vancouver British Columbia Canada; ^3^ Department of Family Practice, Faculty of Medicine University of British Columbia Vancouver British Columbia Canada; ^4^ Department of Family Practice University of British Columbia Vancouver British Columbia Canada; ^5^ Division of General Internal Medicine, Faculty of Medicine University of British Columbia Vancouver British Columbia Canada; ^6^ Centre for Health Education Scholarship and Department of Occupational Science and Occupational Therapy University of British Columbia Vancouver British Columbia Canada; ^7^ Department for Innovation in Medical Education University of Ottawa Ottawa Ontario Canada; ^8^ Office of Faculty Development and Department of Medicine, Faculty of Medicine University of British Columbia Vancouver British Columbia Canada; ^9^ Undergraduate Medical Education and the Division of Critical Care, Department of Medicine, Faculty of Medicine University of British Columbia Vancouver British Columbia Canada

## Abstract

**Background/Purpose:**

Although much has been written about the medical learning environment, the patient, who is the focus of care, is rarely the focus in this literature. The purpose of this study was to explore the role of the patient as an active participant with agency in the medical learning environment from the standpoint of the learner, the attending physician, and most importantly, the patient. We hoped to gain insights into the mechanisms that can reinforce professional values such as patient‐centred and respectful behaviours in a patient‐present learning environment.

**Methods:**

We conducted this study in an ambulatory internal medicine clinic using ‘patient‐present’ clinic visits. All case presentations occurred in examination rooms with the patient. We invited participants (attending physicians, undergraduate and postgraduate learners, patients and family members) to participate in semistructured interviews after each clinic visit to explore the impact of the patient‐present learning environment. We recruited 34 participants in the study; 10 attending physicians, 12 learners, 10 patients and 2 family members. We analysed the data deductively using a conceptual framework of agency.

**Summary/Results:**

We identified three major insights: (1) Patients felt engaged and valued opportunities to be heard; (2) Attending physicians and learners reported a more respectful learning environment and a positive though challenging teaching and learning experience; and (3) A hidden curriculum emerged in a performance‐based view of professional behaviour.

**Conclusions:**

Patient‐present teaching engaged patients and enhanced their agency by recasting the patient as the central focus within the healthcare encounter. We identified a tension between performing and learning. This study adds new insights to the concept of patient centredness and professionalism from the perspectives of all participants in the medical teaching and learning environment.

## Abstract 2

A patient‐centred approach to teaching, learning and patient care is shown to create tension between upholding a performance role versus a learning role that challenges us to think critically about the complexity of balancing needs.

## 1 BACKGROUND

The learning environment has been conceptualised with multiple overlapping dimensions; personal, social, organisational, physical and virtual spaces.^1^ A positive clinical learning environment supports a learner's well‐being, identity formation and performance, whereas a negative one can lead to burnout, fear of uncertainty, medical error and patient harm.^1^, ^2^ In a scoping review of interventions designed to improve the learning environment, exploring the role of the patient in the learning environment was identified as an area for further exploration in the literature.^3^ In recent years, there has been an increasing emphasis on creating and implementing person‐ or patient‐centred care in clinical settings. While approaches vary, they share a common focus on creating an environment that encourages patient engagement.^4^ The clinical context of care is often simultaneously a context in which medical education takes place, suggesting the need to better understand how patient‐centred care also alters the *learning* environment.

Evidence suggests that the patient's presence contributes to a positive learning environment.^3^ For example, some studies have shown that patient inclusion is a strong component of teaching humanistic and professional behaviours to students,^5^ especially when physicians considered excellent teachers can model positive patient care behaviours at the bedside. Further, bringing patient voices into patient‐centred educational experiences as often as possible can mitigate the loss of empathy that occurs in some disciplines with the progression of clinical training.^6^, ^7^ Moreover, a subset of research focusing on bedside teaching shows that, from the perspective of the teacher and learner, creating bedside experiences solidifies the culture of medicine as patient‐centred.^8^, ^9^, ^10^ Bedside teaching allows for greater inclusion of the patient in decision‐making, encourages efficiency in history presentations and evaluations and serves as a reminder of the patient role in education and medicine.^9^, ^11^, ^12^ Patients, too, appear to benefit from bedside experiences, reporting increased understanding of their disease, a greater sense of compassion and respect from the medical team and increased inclusion in their medical care.^13^

We chose the internal medicine ambulatory clinic as a ripe ‘laboratory’ to explore the effect of patient presence during teaching rounds on the quality of education, effect on patient‐centred care and the learning environment. Thus, the purpose of this study was to explore how the patient's presence shapes the care and learning environment for all participants—learner, attending physician and, most importantly, the patient. We consider these insights critical not only to improving patient‐centred care but also in creating positive learning environments where all participants are empowered to present the *best version of themselves*.^14^

## 2 METHODS

Our study team consisted of a citizen‐patient, learners (medical students and residents), physicians and researchers with the aim to advance our understanding of the patient's influence on participants' perceived agency and professional behaviours in the clinical learning environment. The study team met regularly to define and to refine the study question, construct the interview guides using our unique perspectives and discuss and agree on the thematic analysis. The three interviewers were trained in best practices for conducting semistructured interviews^15^ by a study team member with expertise in training interviewers and conducting interviews for research. The interview probes were woven into the interview guides after reviewing previous interviews, with input from all study members.

### 2.1 Study design

We used a design‐based research approach to guide our study, wherein we subjected our interview guides to iterative cycles of planning, testing and refinement.^16^ The study question was to explore how the patient's presence impacts the learning environment and shapes the perception of agency for all participants—learner, attending physician and, most importantly, the patient. We applied the conceptual framework of agency,^17^ the degree to which people feel either free or constrained in a given situation, as our lens to design and conduct interviews. We were interested in the patients' feeling of agency to express their concerns and in the learners' and attending physicians' perception of agency to be their best professional self in the context of a learning environment that included the patient.

### 2.2 Setting

We conducted this study in the Ambulatory Internal Medicine Clinic (AIMC), a teaching clinic, in a clinical academic campus of our university medical school.

### 2.3 Intervention

To explore the role of the patient as an active participant in an authentic clinical learning environment, the patient was present for all teaching and learning experiences in the AIMC encounters studied. Each attending physician committed to conducting patient‐present teaching clinics with one to two patients each day throughout the week. Our intervention entailed having the learner present the entire case to the attending physician in front of the patient; additionally, all ensuing discussion also occurred in front of the patient. Usually, in this clinic, most aspects of the conversation around the case presentation occur in a conference room, hallway or other location sequestered away from the patient. After the clinical teaching encounter, we interviewed each member of the interaction with interview guides tailored to the patient, the learner and the attending physician. Postinterventional interviews proceeded in two ways: (1) with the patients and learners immediately after their appointment and (2) with attending physicians at the end of the week in the AIMC.

### 2.4 Participants

University and health authority behavioural research ethics boards approved this research plan. We recruited three types of participants for the study: attending physicians,^10^ learners (medical students and residents)^12^ and patients^10^ and their family members.^2^ The total sample size was 34 participants. The average age of attending physicians was 41 years, with teaching experience ranging from 4 to 36 years. Of the learners, five were third year medical students and seven were residents with training levels ranging from postgraduate years 2–4. The average age of patients was 50 years old.

We reminded patients that participation was optional and that clinical care would not be compromised if they declined to participate at any point during the study. We invited attending physicians, patients and learners to participate in a voluntary postinterventional interview. We did not inform the attending physicians if any individual declined to participate.

### 2.5 Data collection

We asked participants questions surrounding positive and negative experiences related to the learning environment, changes in behaviours and attitudes of the medical team and overall impact on the learning environment. In accordance with design‐based research processes, our interviews followed an iterative process of design, evaluation and redesign. As we completed interviews, we made iterative modifications to the interview guide, including probing questions. These probing questions were a response to constructs the investigators thought should be asked in a more targeted way or when previously formulated questions did not yield much discussion. Typically, after each set of weekly interviews, we applied amendments to the interview questions for the following week.

### 2.6 Data analysis

The investigator research team was comprised of clinical and academic faculty, educational leads, faculty development leads and a patient researcher. The team met regularly, and all co‐investigators read the first 21 transcribed interviews as they were completed to gain insights into participants' responses prior to analysis and to discuss and share our emerging impressions. Three members of the team then independently coded the transcripts using NVivo™ Version 12 (Doncaster, Australia) according to what investigators commonly agreed were items of interest, such as changes in the teaching and learning experience, experiences of patient engagement and a need to alter language in response to the intervention setting. We employed a deductive analytic approach using a framework of agency. We merged and categorised item level codes into theoretically informed pattern codes.^18^ We then further refined the pattern codes through an iterative analytic process that involved the input of all co‐investigators. After a series of team meetings where iterative discrepancies in coding were discussed and negotiated, the research team reached consensus on dividing the pattern codes into three broad themes: enhanced patient centredness, a challenging learning environment and a performance‐based view of professionalism.^19^ Analysis proceeded until sufficient insights were reached that had conceptual depth of understanding into the research question.^20^ Data analysis internode agreement exceeded 92% using NVivo software.

## 3 RESULTS

Thirty‐six interviews were conducted with 10 Attending Physicians (one interviewed three times), 12 trainees (6 medical students and 6 residents), 10 patients and 2 family members. The Attending Physicians ranged in age from 33 to 66 and in years of teaching from 4 to 36 years. The trainees ranged in age from 25 to 33 years of age. The patients and one family member ranged in age from 33 to 93 years of age, with an equal distribution of male and female participants. The patients were from various self‐reported ethnicities and first languages other than English, including Javanese, Chinese, Indigenous, Filipino, Romanian and Portuguese. All participants were sufficiently fluent in English, and interpreters were not required.

We identified three overarching themes in the reports of attending physicians, learners, and patients. The first theme was enhanced patient centredness. This theme suggested the patient‐present teaching model allowed for an inclusive and patient‐centred health care environment. The second theme was a challenging learning environment, meaning that attending physicians and learners reported that presenting cases with the patient‐present created complexity in balancing patient care with teaching. The third theme was a performance‐based view of professionalism as participants navigated their roles in the altered learning environment. Each theme is described through the lens of agency in the sections that follow, in terms of whether participants felt empowered or constrained to present the best version of themselves. Tables 1, 2, 3 outline the three key themes and their supporting nodes and exemplary participant quotes.

**TABLE 1:** Theme 1: enhancement of patient centredness

Node	Representative quotes
Patient centredness	**Attending physician:** ‘In terms of patient‐centred approach, it comes down to mostly allowing the patients to explain why they are here and give them opportunity to speak’.
	**Trainee:** ‘You want to make sure the patient feels involved in their care, so understands what their conditions are, their pre‐existing conditions, understands what medications there are, and understands how seeing us today is going to change that trajectory one way or the other’.
	**Patient:** ‘The time that they take to understand your symptoms and asking the follow‐up questions. Obviously not just rushing you out the room. I think that is the main thing for me. And then communicating it back as to what they believe your symptoms are. Not to also scare you, in their initial diagnosis as well’.
Engages patient	**Attending physician:** ‘I think the other thing is that it just builds a lot of cred with the patient so if they see you, if they hear everything that they have said kind of summarized and they see you listening attentively and thinking and asking more questions, they feel that you have actually spent time with them and they feel listened and you create a bond with them’.
	**Trainee:** ‘It might be nice for the patient just to hear their whole story repeated back to them because then they know for sure that the story that is being relayed to my staff is exactly the story that they have relayed to me, and they have this extra opportunity to be involved, if they wanted it, as I'm telling the story, you know, to jump in and add something that they may not have thought to add the first time around, or if I get anything differently than what they intended it to be. Then they have the chance to rectify as we go along’.
	**Patient:** ‘It definitely put me at ease, just being able to see and hear what they had to say as they were hearing the medical history. I mean you always get nervous when the doctors leave the room and have to talk among themselves. It definitely put me at ease having that discussion in front of me, especially since they can get the medical history right’.
Patient‐present but not engaged	**Attending physician:** ‘I was mindful that the patient and their family, listening to all that jargon, would not find the additional information useful. I did not want them to be disengaged from the meeting because I felt it was more collaborative’.
	**Trainee:** “I think sometimes the patient might feel excluded in those moments, like ‘what's going on?’ if we are talking about something more technical. Again, it depends on the patient's level of comfort”

**TABLE 2:** Theme 2: a challenging learning environment

Node	Representative quotes
Better learning and teaching experience	**Attending physician:** ‘You actually learn to use more colloquial terms and address things that the patient might understand more. So, I think it might help you in terms of using less medical jargon and being able to communicate in a way that is more understandable to the patient’.
	**Trainee:** ‘At the same time I also learn the communication skills it takes to reassure patients, especially the worry, and you know, just learning about communication techniques from that’.
	**Patient:** “And, you know, suggesting, I do not want to say testing, but just teaching, referring to other tests, other suggestions so that the resident can learn or maybe respond saying ‘no, you do not need that test’ or ‘I did not think that test was necessary because of X, Y, Z.’ So I thought that was very good for a teaching method for the student and the doctor.”
Negative learning and teaching experience	**Attending physician:** ‘I think I would have probably gone into more detail about different guidelines or evidence‐based approaches … But I was mindful that the patient and their family, listening to all that jargon, would not find the additional information useful. I did not want them to be disengaged … I felt like I tried to engage the patient more, but at the sacrifice of the teaching points I would normally make’ and ‘I might be perhaps less aggressive about not necessarily pimping but testing a resident's knowledge of something in front of the patient because I would not want to embarrass him or her’.
	**Trainee:** ‘Maybe you'd spend a little more time talking though clinical reasoning and what your differential is and what your management plan is whereas if you are in front of the patient, you are a little more prone to cut to the chase’.
Performance anxiety and management	**Attending physician:** “I find, with especially junior trainees … it's more of a comfortable environment as well, to just discuss things outside the room because they feel more comfortable in terms of saying ‘I do not know what this means’. I think there is a decent amount of performance anxiety when they discuss these results in front of patients.”
	**Trainee:** ‘I think maybe, if a learner is at a very junior stage, this can be a little more challenging because there may be a lot of things that they want to confirm with the supervisor as they are telling the story. But, typically the patients are aware too, that the learner is at a very junior stage, so they tend to be more patient in that perspective as well’
Patient factors	**Attending physician:** ‘It works well for a subset of patients if there are straightforward identifiable medical issues that can be discussed in front of the patients, that's fine. Maybe some patients with more somatic complaints and may not be an easily recognizable diagnosis can be more challenging’
	**Trainee:** ‘I think it depends on if there are somethings that may be difficult for the patient to hear, I would probably bring that up separately with the attending before I go in. For instance, if the patient is likely to suffer from a factitious disorder’

**TABLE 3:** Theme 3: a performance‐based view of professionalism

Nodes	Representative quotes
Professionalism empowers	**Attending physician:** ‘The patient is looking at you listening to the story. So your body language and your posture, and I think everything is just a little more activated than it might be if, you know, you were listening to the case in private, so to speak, where you are kind of focusing on different things than when you are the other, quote unquote, filler. You may not necessarily be as engaged’.
	**Trainee:** ‘But here, I guess you are thinking more that the patient is in the room, so you are sort of phrasing things and speaking a certain way that would include them in the conversation, rather than shop talk between two physicians’.
Professionalism constrains	**Attending physician:** ‘I like to give my students an opportunity to tell me what they want to do because I want them to try to commit to some clinical decisions, even if they are totally wrong, and I'm totally fine with that because I just want to see where their clinical reasoning is. But I do not think that that's appropriate to do in front of the patient’.
	**Trainee:** ‘The clarity of the thought process was compromised as a result of being distracted and not wanting to upset the patient … the execution of the approach changed by being scattered and disorganized … It was harder. It was definitely harder for the same reasons. I wasn't my best professional self’.
Performance anxiety and management	**Attending physician:** ‘It's nice to challenge a learner and understand why they are coming up with this particular thought process and you do not want to demean or have the patients lose trust in your trainee when they miss something, or you do not agree with their assessment’.
	**Trainee:** ‘It definitively made it more anxiety‐provoking and made me question my competence in attaining respect by the staff’ and ‘It might also give the impression to your attending or preceptor that you do not really know the medical terminology’,

### 3.1 Enhanced patient centredness

We found a strongly repeating narrative of patient engagement and patient centredness from the patients, attending physicians and learners. When asked what patient centredness meant to the three groups of stakeholders, there was overwhelming consensus that it meant having the patients feel heard, included and confident in understanding their medical care.

Of the 36 interviews conducted, 33 interviewees spoke positively about patient engagement in the patient‐present teaching model. From the attending physicians' perspective, increased personal interaction with patients led to an impression of increased engagement. They explained that patients may feel disengaged when left waiting while conversations about them are occurring elsewhere. They perceived that patient presence allowed patients to contribute more to the conversation by elaborating on details of their history as conveyed by the learner. Learners felt very similarly to the attending physicians and echoed that they perceived this approach allowed for patients to have a clearer picture and understanding of their medical diagnoses and necessary treatment plans. They described the importance of patients hearing the detailed thought process behind certain treatment plans.

At the same time, participants also reported concerns about possible patient disengagement. Specifically, attending physicians and learners were concerned the patient would feel lost and less engaged due to medical jargon, the academic level of conversation and the length of review. They were also cautious about worrying the patient with broad differential diagnoses that could allude to serious but less likely conditions such as cancer.

The concerns regarding patient disengagement were not reiterated by the patients. In contrast, patients contributed an overwhelmingly positive response to this teaching model. They reported a high level of engagement in their visit by witnessing the medical thought process, even if they could not understand it all. They appreciated learning *why* changes were being made to their medications, for example, instead of being simply instructed to take an altered dose. Among the patient interviews, only one patient expressed feeling disengaged by the medical jargon used; the others had no complaints in this regard. Overwhelmingly, patients felt reassured in hearing the information they had relayed to the learner later translated to the attending physician. Patients indicated confidence that they were heard by the learner and the attending physician. They also appreciated being able to correct or add details to the ongoing conversation. Ultimately, for the patients in this study, a patient‐present teaching model reinforced a more patient‐centred and inclusive health care environment.

### 3.2 A challenging learning environment

A negative impact on teaching and learning emerged as performance anxiety and management in seven attending physician interviews and eight learner interviews. Concern about a negative impact on teaching and learning largely came from the perspective of attending physicians. They indicated that they hesitated to adopt very detailed, evidence‐based teaching for fear of alienating the patients with their medical jargon or increased length of the visit. They also worried about probing for fear of making learners look incompetent in front of the patient. Additionally, they felt that patient‐present teaching might create a less safe environment for learners to ask questions. Learners expressed similar barriers to discussing detailed teaching points and asking questions in the room with the patient‐present. To mitigate these barriers, 13 interviewees (8 attending physicians and 5 learners) suggested preparing the learner in advance and/or debriefing after the interaction.

An increase in performance anxiety that correlated to a learner's year of education was also reported. Junior learners (third or fourth year medical students), who are at the stage of becoming familiar with the foundations of medicine, said they felt that they not only had to impress their attending physician but also had to continue to engage with the patient in the room. According to some learners, this perceived balancing act made for a more challenging learning environment.

In contrast, 15 interviewees (8 attending physicians, 4 learners, 2 patients and 1 family member) described the patient‐present teaching model as leading to a high‐quality teaching and learning experience. This positive impact was largely attributed to being able to teach and assess students in real‐time and the opportunity to coach crucial skills such as patient interaction and communication.

The last identified barrier to patient‐present teaching related to patient‐specific characteristics. Learners and attending physicians noted it would be difficult to discuss sensitive topics such as substance use or psychiatric diagnoses. They indicated that individual patient attributes also played a role with, for example, patients who interrupt often potentially impeding the teaching exchanges. Overall, attending physicians and learners reported that, in trying to navigate the relations among all three stakeholders, having the patient‐present created challenges to robust teaching and learning.

### 3.3 A performance‐based view of professionalism

When questioned about perceptions of professionalism, attending and learner interviewees suggested that patient presence both empowered and constrained them. On the one hand, patient presence was seen to contribute to an environment that encouraged participants to be ‘more professional’. Attending physicians and learners commented that patient‐present teaching helped discourage inappropriate language and comments about patients. They described an increased formality of interaction between attending physician and learner. On the other hand, attending physicians and learners often felt it was uncomfortable to probe a learner's knowledge in front of patients. This was due to an anticipation that it would undermine patients' confidence and increase anxiety of learners. Similarly, learners feared appearing incompetent to both the attending physician and the patient. When presenting the case, they felt it was challenging to balance medical terminology for the attending physician with lay language for the patient and that this balance at times led to greater disorganisation in the presentation. Learners also worried about presenting a plan in front of patients that they had not previously discussed with the attending physician for fear it could be wrong.

## 4 DISCUSSION

Evidence has shown patient inclusion in medical education not only improves learners' perceptions of patient‐centred care but also increases empathy in clinical training.^21^ Yet, a clear understanding of the patient's role and its effect on the learning environment is lacking. Through a closer look at the patient, learner and attending physician experience in an authentic clinical environment, we began to see the reciprocal effects each role had on the learning environment. Anthony Giddens' lens of agency remind us that social structures and individuals exist in an intimate, *mutually influential* relationship; social structures are created and recreated by individuals in an environment, while individuals also rely on rules and resources of the social structures to determine their action.^17^ While agency is usually rooted in the ability to present your most authentic self, in this study, we applied the construct of agency to professionalism, the extent to which participants are free to the *best version of themselves*.^14^

Previous studies have examined the role of the patient inclusion in bedside rounds but most examined from the perspective of the attending physician and the learner^22^, ^23^ and some surveyed patients as well.^24^, ^25^ This study is the first to explore, through in‐depth interviews, the perspectives of the patient, the learner and the attending physician, illuminating the reciprocal effects each role had on the learning environment. In contrast to the previous in‐patient studies, this study took place in an outpatient or ambulatory setting.

### 4.1 Patient centredness

Patient centredness is a cornerstone of current day medicine, as evidence by a growing body of recommendations.^26^ We were specifically interested in participants' own concepts of patient centredness, and indeed, members of all three participant groups described a similar understanding of patient centredness: ensuring that the patient feels heard, included and confident in understanding their medical care. We conceptualised patient centredness as creating an environment that elevates the patient's agency in a clinical setting by allowing them to assert themselves more effectively. When patient‐present *teaching* occurs, it appears to enable patient's agency by allowing space to correct and clarify their history and to ask questions that clarify changes to their health care. Yet, although this model supports increased patient agency, the medical professionals reported being constrained by concerns that teaching was burdensome, esoteric or overwhelming to the patient. However, patients wanted to maximise their exposure to conversations about their health, even if they were not directly engaged by the speakers and even struggled to follow all that was said. It appears that in our study, physicians acted on the basis of empathetic assumptions about how they perceived patients and learners to feel. Similarly, learners anticipated what both the attending physicians and patients expected of them and then modified their actions based on these sometimes‐conflicting assumptions of how their role will be seen. There was a consensus among all groups of participants that patient‐present teaching increased patient centredness as they understood the concept. Even so, some attending physicians remained somewhat hesitant to adopt and integrate this model of teaching as routine practice.

### 4.2 Challenging learning environment

The addition of the patient into the clinical teaching environment resulted in a degree of challenge for both the attending physicians and the learners. Framing the environment using a dramaturgical metaphor^27^ can help us better understand why this might be. Whereas, in the patient‐absent environment, the attending physician is clearly the audience for the learner's performance of the case presentation, in the patient‐present environment, things are not so straightforward. The patient becomes a second audience, watching the traditionally ‘backstage’ interaction between the attending physician and the learner now being performed ‘frontstage’. The attending physician's performance as teacher is now not just for the learner, who has some medical knowledge and is there to be taught, but also for the patient, who is not a medical insider and who is not there to be trained as one. The fact that the patient is there to receive treatment from a caring and competent expert compounds the challenge, as the roles of both attending physician and learner are, of course, dual (i.e., they are teacher and trainee but also both care providers to the patient). Hence, attending physicians expressed concerns over in‐depth teaching and/or correction that might bore or exclude the patient or else make the patient feel uncertain about the learner's competence as care provider. Moreover, they worried that teaching and correction in front of a patient might also threaten the learner's face (or self‐image) and/or create a more tense environment for learners performing already high‐stakes presentations. Learners, too, expressed confusion resulting from the addition of the patient as audience. They were unsure which took priority: impressing their attending physician as learners with their insider knowledge, or including the patient as care providers, which they felt was difficult to do through a formal case presentation script replete with medical terminology.

Instead of a two‐way interaction, then, this suddenly became a triad of individuals whose roles changed, dependent on the social performative interplay occurring in the room. This reciprocity demonstrates the organic changes in agency that occur, as well, when there are more persons in an interaction. It was perceived that the attending physician's agency to teach without fear of isolating the patient or demeaning a student, and the learner's agency to ask questions and learn without fear of judgement, would be constrained by the patient. This resonates with Anthony Giddens' theory of agency, where our ability to exert ourselves changes depending on a set of social structures. Sebok‐Syer et al.^28^ explore how patients are positioned in the clinical triad and also conclude that patient involvement in the assessment design gives agency to the patient and links patient outcomes and educational outcomes. Ultimately, we see that although *patient‐present teaching* did create a patient‐centred and inclusive environment, the merging of front‐ and backstage scripts causes a confusion of roles. Thus, this fosters a challenging educational environment to navigate for the attending physician and learner.

### 4.3 Performance‐based professionalism

Medical educators often intend to impart their professional values to the next generation through role modelling.^29^, ^30^ We set out to see if attending physician and learner perceived their professionalism shifted in terms of actions and behaviours towards patients, when patients were present in situations in which they normally do not take part. However, what we discovered was a performance‐based view of professionalism. Professionalism was viewed not only as appropriate interactions with patients but also as competence in demonstrating biomedical expertise. This could be understandable from the viewpoint of the physician: Being knowledgeable and competent as a biomedical expert is an integral professional expectation. Yet, when analysed through the lens of a learner, it may be difficult to know how to strike the right balance between the reality of their not‐yet‐expert status and the expectation that part of their training is to perform as if they were knowledgeable and competent experts.^31^ One of the common concerns of attending physicians with a patient‐present teaching model was unintentionally undermining the learner's performance as an expert through teaching in front of the patient. On the one hand, attending physicians and learners commented that patient‐present teaching helped discourage inappropriate language and comments about patients. On the other hand, however, attending physicians and learners often felt it was unprofessional to question learner competence in front of patients because they perceived this would decrease the patient's confidence in their providers. Specifically, attending physicians did not want to make their learners ‘look bad’ in front of a patient by probing them with questions they might answer incorrectly.

As for the learners, they feared appearing incompetent to both the attending physician and the patient. When presenting to the attending physician, they describe how it was challenging to balance medical terminology for the attending physician with lay language for the patient. They reported that they worried about appearing incompetent to the attending physician because they used fewer medical terms or because they were unorganised in their delivery of presentation. Learners also worried about presenting a plan in front of patients that had not been previously discussed with the attending physician for fear it could be wrong. Goffman's dramaturgy theory^27^ is, again, useful in providing a framework with which to understand this tension, as one of its key tenets is that social interactions, in any given environment, are viewed as performances by actors.^32^ People act both on a front stage, where there are the intentional actions for an audience, and on a backstage, where actions are hidden from an audience. Within a clinical environment, attending physicians and learners could be seen as actors on a ‘professional’ stage ‘performing’ for the audience, the patients. Here, we argue that all stakeholders (i.e., the attending physician, learner and patient) perceive new performance expectations in the changed geometry of a mutual learning triad. This is especially true from the perspective of the attending physician and learner as they are seen as the professionals in the room. These conversations suggest a hidden curriculum where expectations of ‘performing’ underlies the explicit purpose of the encounter as a teaching experience.

## 5 LIMITATIONS

The purpose of this study was not to compare or contrast different models of including the patient in the learning environment; we explored the effect of patient‐present teaching on perceptions of agency with respect to professional behaviours. As such, we sampled the subset of interactions consisting of patient‐present teaching encounters in an ambulatory clinic. Participants in other types of teaching interactions were not interviewed for comparison purposes, and the patients we interviewed would not necessarily have alternative experiences to compare with. The interviews were conducted after the encounter, which was not directly observed by the researchers, and depended on the recall of the participants. The strength of this approach was that all participants in the learning triad were interviewed, eliciting their individual perspectives. Finally, we acknowledge that this study was undertaken in a single outpatient clinical environment. Therefore, although these findings may translate to an in‐patient setting, unique challenges may arise in another setting.

## 6 CONCLUSIONS AND IMPLICATIONS

In this study, participants reported that patient‐present teaching empowered patient health care interactions by increasing engagement of the patient. We found that despite the fear of overwhelming a patient with medical jargon, conversations were well‐received and welcomed by patients. Additionally, this learning context recast the patient as the central focus for learners and attending physicians. Importantly, the attending physicians reported on the positive impact of being able to teach and assess students in real‐time, providing much needed opportunities to directly observe learner‐patient interactions. Yet, patient inclusion limited perceptions of agency in teaching and learning for the attending physician and learner by creating a tension between upholding a performance role versus a learning role. This key insight challenges us to think critically about the complexities of balancing the role of the learner as both a performer and as a learner.

Further research can continue to explore the concepts of performance versus learning as they emerge in‐patient‐present learning. Specifically, we could explore the extent to which patients, physicians and learners regard ‘professionalism’ as synonymous with mastery of biomedical expertise. If the teacher, learner and patient all have a shared expectations of the learning goals, would that change learners' perceptions and anxieties about seeming to ‘fail’ in front of the patient? In this sense, we may be preventing impactful teachable moments out of our desire to maintain an outward appearance of ‘always being right’, regardless of whether patients themselves rely on this expectation. Furthermore, it is unclear whether these attitudes from teachers and learners persist even when patients do not share this perspective.

To conclude, we observed that patient‐present teaching supported patient empowerment and patient centredness in the medical learning environment with the following considerations for the attending physician, the learner and the patient: (1) Reassure the attending physician that *patient‐present teaching* empowers the patient without compromising their experience. Prepare the attending physician to address the student's performance anxiety and consider debriefing sensitive issues outside the patient‐present interaction; (2) Assess the readiness of the learner: The more experience and confidence the learner has, the less anxiety‐provoking is this new setting. Prepare and debrief the learner (describing the teaching approach before going in and asking whether they have any questions after the encounter) to mitigate fears of failing in front of the patient; (3) Partner with the patient for a positive learning experience. Establish a shared understanding of the goals and expectations of the encounter. Inform the patient that medical language will be used for the benefit of the learner and that such use of language is not meant to exclude them. Finally, thank the patient for helping to put the patient at the centre of learning.

## Supporting information


**Data S1.** Supporting Informtion

## References

[1] Gruppen LD , Irby DM , Durning SJ , Maggio LA . Conceptualizing learning environments in the health professions. Acad Med. 2019;94(7):969‐974. 30870148 10.1097/ACM.0000000000002702

[2] Benbassat J . Undesirable features of the medical learning environment: A narrative review of the literature. Adv Health Sci Educ Theory Pract. 2013;18(3):527‐536. 22760724 10.1007/s10459-012-9389-5

[3] Gruppen LD , Irby D , Durning SJ , Maggio LA . Interventions designed to improve the learning environment in the health professions: A scoping review. MedEdPublish. 2018;7(3):1‐32. 10.15694/mep.2018.0000211.1 PMC10701832 38074598

[4] Hudon C , Fortin M , Haggerty JL , Lambert M , Poitras ME . Measuring patients' perceptions of patient‐centered care: A systematic review of tools for family medicine. Ann Fam Med. 2011;9(2):155‐164. 21403143 10.1370/afm.1226 PMC3056864

[5] Weissmann PF , Branch WT , Gracey CF , Haidet P , Frankel RM . Role modeling humanistic behavior: Learning bedside manner from the experts. Acad Med. 2006;81(7):661‐667. 16799294 10.1097/01.ACM.0000232423.81299.fe

[6] Holmes CL , Miller H , Regehr G . (Almost) forgetting to care: An unanticipated source of empathy loss in clerkship. Med Educ. 2017;51(7):732‐739. 10.1111/medu.13344 28892175

[7] Schrewe B , Bates J , Pratt D , Ruitenberg CW , McKellin WH . The Big D (eal): Professional identity through discursive constructions of ‘patient’. Med Educ. 2017;51(6):656‐668. 28488302 10.1111/medu.13299

[8] Reilly JB , Bennett N , Fosnocht K , et al. Redesigning rounds: Towards a more purposeful approach to inpatient teaching and learning. Acad Med. 2015;90(4):450‐453. 25426739 10.1097/ACM.0000000000000579

[9] Bennett NL , Flesch JD , Cronholm P , Reilly JB , Ende J . Bringing rounds back to the patient: A one‐year evaluation of the chiefs' service model for inpatient teaching. Acad Med. 2017;92(4):528‐536. 28351066 10.1097/ACM.0000000000001459

[10] Gonzalo JD , Heist BS , Duffy BL , et al. The art of bedside rounds: A multi‐center qualitative study of strategies used by experienced bedside teachers. J Gen Intern Med. 2013;28(3):412‐420. 23129164 10.1007/s11606-012-2259-2 PMC3579967

[11] Rowland P , Anderson M , Kumagai AK , McMillan S , Sandhu VK , Langlois S . Patient involvement in health professionals' education: A meta‐narrative review. Adv Health Sci Educ Theory Pract. 2018;24(3):595‐617. 10.1007/s10459-018-9857-7 30306292

[12] Rowland P , MacKinnon KR , McNaughton N . Patient involvement in medical education: To what problem is engagement the solution? Med Educ. 2021;55(1):37‐44. 32350875 10.1111/medu.14200

[13] Lichstein PR , Atkinson HH . Patient‐centered bedside rounds and the clinical examination. Med Clin North Am. 2018;102(3):509‐519. 29650072 10.1016/j.mcna.2017.12.012

[14] Holmes CL , Harris IB , Schwartz AJ , Regehr G . Harnessing the hidden curriculum: A four‐step approach to developing and reinforcing reflective competencies in medical clinical clerkship. Adv Health Sci Educ Theory Pract. 2015;20(5):1355‐1370. 10.1007/s10459-014-9558-9 25319835

[15] DeJonckheere M , Vaughn LM . Semistructured interviewing in primary care research: A balance of relationship and rigour. Fam Med Community Health. 2019;7(2):e000057. 10.1136/fmch-2018-000057 32148704 PMC6910737

[16] Dolmans DH , Tigelaar D . Building bridges between theory and practice in medical education using a design‐based research approach: AMEE Guide No. 60. Med Teach. 2012;34(1):1‐10. 22250671 10.3109/0142159X.2011.595437

[17] Varpio L , Aschenbrener C , Bates J . Tackling wicked problems: How theories of agency can provide new insights. Med Educ. 2017;51(4):353‐365. 28164372 10.1111/medu.13160

[18] LeCompte MD , Schensul JJ . Data analysis: How ethnographers make sense of their data. In: LeCompte MD , Schensul JJ , eds. Designing and Conducting Ethnographic Research. UK: Rowman Altamira; 1999:147‐159.

[19] Braun V , Clarke V . Using thematic analysis in psychology. Qual Res Psychol. 2006;3(2):77‐101.

[20] Varpio L , Ajjawi R , Monrouxe LV , O'Brien BC , Rees CE . Shedding the cobra effect: Problematising thematic emergence, triangulation, saturation and member checking. Med Educ. 2017;51(1):40‐50. 27981658 10.1111/medu.13124

[21] Gaufberg EH , Batalden M , Sands R , Bell SK . The hidden curriculum: What can we learn from third‐year medical student narrative reflections? Acad Med. 2010;85(11):1709‐1716. 20881818 10.1097/ACM.0b013e3181f57899

[22] Merchant NB , Federman DG . Bedside rounds valued but not preferred: Perceptions of internal medicine residents and attending physicians in a diverse academic training program. South Med J. 2017;110(8):531‐537. 28771651 10.14423/SMJ.0000000000000689

[23] Tariq M , Motiwala A , Ali SU , Riaz M , Awan S , Akhter J . The learners' perspective on internal medicine ward rounds: A cross‐sectional study. BMC Med Educ. 2010;10(1):53. 10.1186/1472-6920-10-53 20618929 PMC2912319

[24] Shih AF , Addo‐Tabiri NO , Sofair AN . Patients' perceptions of bedside rounding. South Med J. 2018;111(5):281‐287. 29767220 10.14423/SMJ.0000000000000803

[25] Monash B , Najafi N , Mourad M , et al. Standardized attending rounds to improve the patient experience: A pragmatic cluster randomized controlled trial. J Hosp Med. 2017;12(3):143‐149. 28272589 10.12788/jhm.2694

[26] The Health Foundation . Person‐centred care made simple: What everyone should know about person‐centred care 2016. https://www.health.org.uk/sites/default/files/PersonCentredCareMadeSimple_0.pdf

[27] Goffman E . The Presentation of Self in Everyday Life. Doubleday & Company: Garden City NY; 1959.

[28] Sebok‐Syer SS , Gingerich A , Holmboe ES , Lingard L , Turner DA , Schumacher DJ . Distant and hidden figures: Foregrounding patients in the development, content, and implementation of entrustable professional activities. Acad Med. 2021;96:S76‐S80. 10.1097/ACM.0000000000004094 34183606

[29] Stern DT , Papadakis M . The developing physician—Becoming a professional. N Engl J Med. 2006;355(17):1794‐1799. 17065641 10.1056/NEJMra054783

[30] Benbassat J . Role modeling in medical education: The importance of a reflective imitation. Acad Med. 2014;89(4):550‐554. 24556777 10.1097/ACM.0000000000000189 PMC4885588

[31] Scott IM . Beyond 'driving': The relationship between assessment, performance and learning. Med Educ. 2020;54(1):54‐59. 31452222 10.1111/medu.13935

[32] Monrouxe LV , Rees CE , Bradley P . The construction of patients' involvement in hospital bedside teaching encounters. Qual Health Res. 2009;19(7):918‐930. 19556399 10.1177/1049732309338583

