# Supplementary material for: Patient‐present teaching in the clinic: Effect on agency and professional behaviour
Source: Med Educ. 2021 Sep 6;56(3):270–9. doi: 10.1111/medu.14623 (PMC9292717; doi:10.1111/medu.14623)
Supplement: Supplementary file 1 — Data S1. Supporting Informtion [file MEDU-56-270-s001.pdf]

a place of mind
THE UNIVERSITY OF BRITISH COLUMBIA

Faculty of Medicine
Undergraduate Medical Education
Gordon & Leslie Diamond Health Care Centre
2775 Laurel Street, 11th Floor
Vancouver, BC, Canada V5Z 1M9

Vancouver Coastal Health
Promoting wellness. Ensuring care.

Patient and the Learning Environment Stakeholder Interviews – Question Guide

The purpose of this study is to explore the role of the patient as an active participant with agency in an authentic medical learning environment from the standpoint of the learner, the faculty and most importantly the patient. We hope to gain insight into the reinforcement of positive professional values such as patient centred behaviours and a respectful environment.

Research Question: Applying the social construct of agency, we hope to learn the extent to which the structure of the Internal Medicine Outpatient Clinic patient rounds simultaneously empowers and constrains the participant's ability to be the best professional version of themselves.

This interview guide will take an appreciative inquiry approach.

Learner Interview Questions:

> Note: Probe for more on "rules" around what a medical consultation and learning situation is supposed to be.
>
> e.g., Why isn't that appropriate?
> Where did you learn that "rule"?
> How would you change things to fit the rule?

1. The clinic you were in today adopted an approach to teaching whereby you presented the case and had the discussion in front of the patient. Did you feel this clinical teaching was different from other out-patient rotations you have had? Please explain.
    a. How did that make you feel?
    b. Did you like that better?
    c. How did your approach change to patient presentation?
        i. How did you feel your behaviour was different with the patient present within the learning environment?
        ii. Did your language, demeanour, etc. change because the patient was there?
    d. Did your level of detail change based on your interaction with the patient and/or gestalt about patient preferences/attitudes?

2. Do you feel that this clinical teaching was more patient-centred than other out-patient clinical teaching you have had?
    a. What do you believe patient-centred teaching looks like?
    b. What made this interaction more/less patient-centred than in the past?
3. In this setting, did you feel the learning environment was more or less professional in relation to both the patient and your colleagues?
    a. Was it easier or harder to be your best professional self?
    b. How would you define professionalism for yourself?
    c. Do you think this approach leads to a more respectful learning environment? If so, how and why?
        i. "Have you ever experienced a doctor using inappropriate language to describe a patient, when the patient was not there?"
        ii. "If so, can you describe the experience or tell me what happened? How do you think this model of teaching might impact situations like that?
        iii. Do you think this model of teaching helps mitigate that?"
        iv. " If not, do you think this model promotes a more respectful environment, than if the patient was absent?"
4. What can you and your team do to help your patient feel more empowered and included in the health care plan?
5. What are barriers to this type of set-up?
6. Do you envision this model being sustainable across a variety of clinical settings?

Patient Interview Questions:

1. Today your visit has involved everyone participating in your care. Can you tell me how that felt for you?
    a. What aspect of the encounter, specifically, contributed to this feeling?
    b. How was this experience for you, compared to when the learner isn't present?

    Follow Up Questions to #1:

    c. How do you think this visit changed your understanding of your diagnosis or treatment plan?
        i. How did you feel this information was communicated to you, given the circumstances?
    d. How did this help you or get in the way of asking your questions?
    e. Did you appreciate hearing all of the discussion related to your care?
2. What matters most about your care from medical professionals for your health and well-being? How well do you think your team understood that?
3. Did you feel this encounter was more "patient-centred" compared to previous interactions within the health care system?
4. Tell me whether you felt respected and included today. What made you feel that way?
    a. Were there moments during your visit that you felt excluded from the conversations of your health care team? How did you feel about it?
        i. Would you had preferred if those conversations to happen outside of your room?
5. Was there anything that made you feel uncomfortable today? Please tell me about it.
6. What would have made today's encounter better?
7. Is there anything else you would like me to know about your experience today?
8. Do you think this approach leads to a more respectful workplace? How? Why?

Note: Probe for more on “rules” around what a medical consultation and learning situation is supposed to be.

e.g., Why isn’t that appropriate?
Where did you learn that “rule”?
How would you change things to fit the rule?

Attending Physician Interview Questions:

Physician who previously participated:

Is there anything that you are doing differently as a result of participating in the study? (i.e., having experienced the patient present when discussing with the learner) [looking for long term impact]

New physician participants:

1. This week, you adopted an approach where the case presentation and discussion occurred in front of the patient. What is your normal practice and how did this intervention change that?
2. How did you decide which patients were appropriate to have present for the case presentation and discussion with the learner?
   a. What facilitates your decision? What prevents you from choosing that patient?
3. How do you think this intervention changed team teaching dynamics in this clinic?
   a. What were positive changes to team dynamics?
   b. Were there any negative effects of this model on team behaviours?
   c. What were some of the challenges that you experienced?
4. When you think of modeling professional behaviours to trainees, did you feel more or less constrained with this model?
   a. Do you think this approach leads to a more respectful workplace? If so, how and why?
      i. “Have you ever experienced your colleagues using inappropriate language to describe a patient, when the patient was not there?”
      ii. "If so, can you describe the experience or tell me what happened? How do you think this model of teaching might impact situations like that?
      iii. Do you think this model of teaching helps mitigate that?”
      iv. “ If not, do you think this model promotes a more respectful environment, than if the patient was absent?”
5. When working on the Internal Medicine Ambulatory Clinic, what qualities and actions make you feel the most patient focused in the clinical context?
6. What do you think your patients need from the team in order to feel more empowered and included in their health care plan?

7. How would you modify this teaching model to make it more patient-centred and beneficial to a positive learning environment?
    a. What practical components to this model need to be addressed in order for this style of teaching to be implemented and sustainable across a variety of clinical settings?
